# Mr. Edwards's Case of Inveterate Scurvy

**Published:** 1806-08

**Authors:** G. F. Edwards

**Affiliations:** Surgeon. 13, Walcot Parade, Bath


### *To the Editors of the Medical and Physical Journal*

GENTLEMEN,

THE following case of inveterate scurvy came under my notice; and as I long wished to give a fair trial to the arsenical solution in such cases, I conceived this to be a favourable opportunity; the result is stated exactly as it occurred, and if my feeble efforts can, in any shape, advance medical science, they will be exerted with that view, submitting with all deference to your better judgment such cases as may appear to carry any importance, as new, either in their nature or cure.

The subject of this case, is the cook of a gentleman in this city, a woman about the age of 50. She has been subject to scurvy many years, which commenced in the palms of her hands, and gradually extended over her hands and arms, and partially in other parts of the body; but the hands and arms appear to be primarily and most materially affected, so much so, as to stiffen and partially contract the joints of the fingers and elbows.

Her mind dwelt so much on the disagreeable nature of the disease, as to occasion her general health to decline; her habit of body was costive, with loss of appetite; symptomatic fever, characterised by a small quick pulse, constant thirst, white tongue, and frequent inclination to vomit. She had tried many regular professional men in this city and elsewhere, as also the use of our baths, and many of the nostrums advertised for the cure of such complaints, but in vain. The disease evidently appeared to gain ground, by rendering her less capable of using her hands and arms. In this deplorable state, she applied to me on the 6th of January last. In the first place I deemed it prudent to detach her mind from so forcibly dwelling on her disease, or rather despairing of relief: after which I diminished the prevailing symptoms of fever, by first ordering an emetic, and afterwards a brisk aperient, as the chylopoetic viscera appeared deranged. The emetic and aperients had the effect of restoring the stomach and bowels to a healthy state; small doses of antimonial powder, joined with nitre, were continued till her health was materially mended.

On the 24th of January I ordered her the mineral solution, beginning with gtt. vj. bis die, in decoctum ulmi, increasing one drop per diem, provided nausea was not caused.

This plan was strictly adhered to, and at the end of a week I saw her, and found that the disease evidently was yielding, and the general health mending, an increase of appetite, and undisturbed sleep. She continued the solution for a month, and had increased the dose to gtt. xxx. bis die; at the end of which time I saw her comparatively well; the blotches in her hands, and different parts of the body, were not only gone, but the flexibility of the joints perfectly restored. Animated by the hope that she should obtain a perfect cure, she was anxious for more of the drops; a continuance was ordered, but not an increase, because I found that her health was perfectly re-established, and the disease nearly eradicated, therefore I judged it prudent not to push the remedy farther.

She continued taking 30 drops till the 14th of May, when she expressed herself deeply indebted to me for her cure, and for the enjoyment of health, which she had not experienced for upwards of 30 years; and I am happy to say, that the skin, even of the parts most severely affected, does not bear a trace of any disease, and that she is perfectly restored to health.

I have frequently given it in tertian intermittents with success, and in cases of scurvy; but of its specific qualities in this kind of eruption I never before was so well satisfied.

The antiscorbutic properties of this mineral claims the attention of medical men, in order to discover its further effects in the cure of such diseases.

It appears to me, that the attention should be first directed to the state of the general health, before the administration of the solution, otherwise, the indiscriminate use without judgment, might not only not answer the intended end, but prove dangerous to life itself.

I am, &c.

13, *Walcot Parade, Bath,*

*June* 8, 1806,

G. F. EDWARDS, Surgeon.

*P.S.* I am happy, in common with some others of your correspondents, in bearing testimony to the superior advantages of nitre over other remedies, in the cure of sphacelus, as stated by the ingenious Dr. Cuming. It is most certain, that no other known remedy is so effectual as this, in arresting putrid diathesis, and correcting fætor frm wounds.

